# Treatment of African children with severe malaria - towards evidence-informed clinical practice using GRADE

**DOI:** 10.1186/1475-2875-10-201

**Published:** 2011-07-21

**Authors:** Nyokabi Musila, Newton Opiyo, Mike English

**Affiliations:** 1 Child and Newborn Health Group, Kenya Medical Research Institute-Wellcome Trust Research Programme, PO Box 43640 - 00100, Nairobi, Kenya; 2 Department of Paediatrics, John Radcliffe Hospital, University of Oxford, Oxford, UK

## Abstract

**Background:**

Severe malaria is a major contributor of deaths in African children up to five years of age. One valuable tool to support health workers in the management of diseases is clinical practice guidelines (CPGs) developed using robust methods. A critical assessment of the World Health Organization (WHO) and Kenyan paediatric malaria treatment guidelines with quinine was undertaken, with a focus on the quality of the evidence and transparency of the shift from evidence to recommendations.

**Methods:**

Systematic reviews of the literature were conducted using the Grading of Recommendations Assessment, Development and Evaluation (GRADE) tool to appraise included studies. The findings were used to evaluate the WHO and Kenyan recommendations for the management of severe childhood malaria.

**Results:**

The WHO 2010 malaria guidance on severe malaria in children, which informed the Kenyan guidelines, only evaluated the evidence on one topic on paediatric care using the GRADE tool. Using the GRADE tool, this work explicitly demonstrated that despite the established use of quinine in the management of paediatric cases of severe malaria for decades, low or very low quality evidence of important outcomes, but not critical outcomes such as mortality, have informed national and international guidance on the paediatric quinine dosing, route of administration and adverse effects.

**Conclusions:**

Despite the foreseeable shift to artesunate as the primary drug for treatment of severe childhood malaria, the findings reported here reflect that the particulars of quinine therapeutics for the management of severe malaria in African children have historically been a neglected research priority. This work supports the application of the GRADE tool to make transparent recommendations and to inform advocacy efforts for a greater research focus in priority areas in paediatric care in Africa and other low-income settings.

## Background

Severe *falciparum *malaria is a medical emergency. An estimated 800,000 annual deaths occur in African countries South of the Sahara as a direct result of malaria in children aged five years and less [1]. The Roll Back Malaria Partnership's goal is to control and ultimately eliminate malaria, which is consistent with the Millennium Development Goals (MDGs) 4, to reduce under-five child mortality, and 6 to reduce the incidence of infectious diseases including malaria. However, until achieved, appropriate treatment of severe malaria to prevent fatality and disability will be required. In spite of its narrow therapeutic window, which dictates careful dosing and monitoring, quinine has been the drug of choice for the treatment of severe malaria in Africa for over 30 years (and before that was in use since the 17^th ^century). Exciting new results from an African multi-centre trial now suggest that artesunate should replace quinine as the first-line treatment [2]. In Kenya, however, changes are anticipated to take place progressively over the coming years as practical issues, such as making changes to national policy, procurement and supply chain issues and re-training of health workers, are tackled. In particular, the latter will demand a significant injection of funds from the national malaria control programme (Personal Communication, Dr Elizabeth Juma, Head of Division of Malaria Control, Ministry of Public Health and Sanitation). Meanwhile, quinine will inevitably remain the default treatment.

Best approaches to treatment are often encompassed in clinical practice guidelines (CPGs), tools to support health workers to make evidence-informed decisions in the management of their patients. Ideally CPGs combine the best available research gathered using standardized, robust, systematic methods and contextual factors, such as cost, feasibility, values, resources [3]. The World Health Organization (WHO), which is tasked with producing global clinical guidance, particularly for developing countries, has recently adopted one such transparent and systematic approach to developing its CPGs. This process applies the Grading of Recommendations Assessment, Development and Evaluation (GRADE) tool to transparently assess the quality of research evidence and develop recommendations [4]. The GRADE approach has rapidly become widely used in full or in part, and is endorsed or used by organizations including WHO, the UK's National Institute for Health and Clinical Excellence (NICE), the Scottish Intercollegiate Guidelines Network (SIGN), and the Cochrane Collaboration [5].

In March 2010, the WHO revised its malaria treatment guidance as a framework for country-level policy makers to adapt. For the first time, it incorporated the GRADE approach [6]. In Kenya, the Division of Malaria Control (DoMC) took the opportunity to revise its national guidelines for treatment of severe malaria in children, making them consistent with the 2010 WHO Malaria treatment guidelines (Table 1). In so doing, it changed Kenya's recommended quinine regimen from one that had been disseminated and promoted for over 10 years. Specific changes were an increase in the loading dose and maintenance doses of quinine. This policy change coincided with efforts by researchers, the Kenyan Ministry of Medical Services and others to update CPGs for management of 12 key paediatric conditions, including malaria [7], an approach also attempting to employ the GRADE tool [8]. However, as the 2010 WHO guidance did not employ the GRADE approach to systematically appraise the evidence available to inform the optimum use of quinine in African children, a series of systematic reviews were conducted to examine the quality of evidence available to help guide use of quinine in this population using the GRADE approach. This work demonstrates how treatment of severe malaria has largely been neglected by the research community. Further, it demonstrates how the GRADE tool helps make this explicit, pointing to its potential value for highlighting failures to invest in research on neglected diseases or populations.

**Table 1:** Summary of World Health Organization (2010) and Government of Kenya (2005 and 2010) clinical practice guidelines for the management of children with severe malaria

Treatment for children aged < 5 years with a diagnosis of severe malaria	GoK 2005 Basic Paediatric Protocols*	GoK 2010 Malaria Case Management Guidelines	WHO 2010 Malaria Case Management Guidelines
Pre-referral quinine loading dose	**15 mg/kg **IM injection	**20 mg/kg **IM injection	**20 mg/kg **IM injection
First-line treatment with quinine dose	**15 mg/kg **loading dose then **10 mg/kg every 12 hours **by IV infusion or divided IM injection	**20 mg/kg **loading dose then **10 mg/kg every 8 hours **by IV infusion or divided IM injection	**20 mg/kg **loading dose then **10 mg/kg every 8 hours **by IV infusion or divided IM injection

*Based on 1998 DoMC Malaria Treatment Guidelines.

## Methods

Key clinical questions to be tackled in a series of systematic reviews that addressed the themes of effectiveness and safety of quinine for the treatment of Kenyan children with severe malaria were identified. These were:

1) Systematic review 1: Is there a value in administration of a loading dose of quinine in African children with severe malaria?

2) Systematic review 2: Should Kenya change its recommendation for treatment of severe malaria in children under 5 of 15 mg/kg loading dose followed by 10 mg/kg every 12 hours and replace it with the WHO recommended regimen of 20 mg/kg loading dose followed by 10 mg/kg every 8 hours?

3) Systematic review 3: What are the pharmacokinetics and effectiveness of IV-administered quinine compared to IM-administered quinine in African children with severe malaria?

4) Systematic review 4: Is there a link between IV-administered quinine and risk of hypoglycaemia in African children with severe malaria?

The clinical questions were developed in a PICO (Population, Intervention, Control/Comparator, Outcome) format and literature searches conducted in PubMed and the Cochrane Library (up to September 2010) using MeSH (Medical Subject Headings) search terms derived from PubMed. Free text searches with no language or time limitations were conducted. Additional searches were also conducted in PubMed using the clinical queries filter tool. Furthermore, bibliographies in the WHO guidelines and abstracts of the 5th Multilateral Initiative on Malaria Pan-African Malaria Conference (2-6 November 2009, Nairobi, Kenya) were scanned. In a first round, identified manuscript titles were independently screened by two investigators (NM, ME) using pre-defined inclusion/exclusion criteria (Table 2). In a second round of screening, abstracts and full texts were read to select relevant studies to be included in the systematic reviews. Data from included studies were extracted into an in-house data extraction tool. Quinine doses were uniformly presented in the salt form as quinine sulphate. The GRADE tool was then used to critically appraise the quality of each study and following that, the quality of all available evidence informing a particular question/outcome combination was categorized based on GRADE guidelines (Table 3). Where no evidence of heterogeneity was found, binary outcome data from multiple studies were pooled in a random-effects model using STATA 11 (STATA Corporation, College Station, Texas, USA), and assessed for heterogeneity using the I-Squared test.

**Table 2:** Study selection inclusion/exclusion criteria

Inclusion criteria	Exclusion criteria
*P. falciparum *malaria	Uncomplicated malaria
Randomized Controlled Trials (observational studies to be considered if no RCT evidence)	IR administration
Children < 5 years old (older children and adult data to be considered if no studies in children)	
Diagnosis of severe or complicated malaria, confirmed with microscopy or rapid diagnostic test or cerebral malaria	
African setting	
IM or IV administration	

**Table 3:** Evidence quality and level of confidence in estimates using the GRADE approach

Evidence Quality	Level of confidence in estimate
High	Further research is ***very unlikely ***to change our confidence in the estimate of effect
Moderate	Further research is ***likely ***to change our confidence in the estimate of effect, and may change the estimate
Low	Further research is ***very likely ***to change the estimate of effect, and is likely to change the estimate
Very low	The estimate of effect is very uncertain

Adapted from Guyatt et al 2008 [26]

## Results

The number of studies identified in the literature search for each question and then the number screened for eligibility and final included studies in the respective systematic reviews are presented in a series of flow diagrams (see Additional File 1).

### Systematic review 1: Is there a value in administration of a loading dose of quinine in African children with severe malaria?

One Cochrane systematic review that was a meta-analysis of four African randomized controlled trials was identified [9] (Table 4). Moderate to low quality evidence for critical and important outcomes indicates that a quinine loading dose of 19 to 21 mg/kg may make little or no difference to mortality, coma recovery time, convulsion frequency, asexual parasitaemia at 24 and 48 hours, neurological sequelae or adverse events including hypogylcaemia, hypotension and arrhythmia. However, moderate quality evidence indicates that a loading dose of 20 mg/kg quinine may reduce fever clearance time [Weighted Mean Difference (WMD) 11.11, 95% Confidence Interval (CI) 20.04 to 2.18; n = 68] and parasite clearance time (WMD 7.44, 95% CI 13.24 to 1.64; n = 67) as well as cause temporary hearing loss [Risk Ratio (RR) 3.14, 95% CI 1.05 to 9.38; n = 33] (see Additional File 2).

**Table 4:** Characteristics of studies that evaluated quinine loading dose (Systematic review 1)

			Test quinine sulphate dose (IV)		Comparator quinine sulphate dose (IV)
Study	Age range	Country	Loading dose	Maintenance dose	Uniform dose
Assimadi 2002* (N = 72)	8 months to 15 years	Benin	21 mg/kg	10.5 mg/kg every 12 hours	15.9 mg/kg every 12 hours
Fargier 1991* (N = 20)	15 years and above	Cameroon	19 mg/kg	9.7 mg/kg every 8 hours	9.7 mg/kg every 8 hours
Pasvol 1991**^# ^**(N = 59)	up to 12 years	Kenya	20 mg/kg	10 mg/kg every 12 hours	5 to 10 mg/kg every 12 hours
Tombe 1992 (N = 33)	14 years and above	Kenya	20 mg/kg	10 mg/kg every 8 hours	10 mg/kg every 8 hours

*Reported quinine doses converted from quinine base to quinine salt using salt conversion factor of 1.21

**^#^**N = 20 patients who received IM quinine were excluded from this analysis

Adapted from Lesi et al 2004 Cochrane systematic review [9]

### Systematic review 2: Should Kenya change its recommendation for treatment of severe malaria in children under 5 of 15 mg/kg loading dose followed by 10 mg/kg every 12 hours and replace it with the WHO recommended regimen of 20 mg/kg loading dose followed by 10 mg/kg every 8 hours?

There were no head to head comparisons or suitable indirect comparison studies identified in the literature search.

### Systematic review 3: What are the pharmacokinetics and effectiveness of IV-administered quinine compared to IM-administered quinine in African children with severe malaria?

Seven studies in African children that evaluated quinine administered with a loading dose by the IV or IM route were identified [10-16]. In addition, three studies identified by a Cochrane review but on African children not strictly having severe malaria were included [17]. These studies compared the effectiveness of quinine administered with a loading dose by the rectal (IR) and the IV route [18,19] and the IR and the IM route [20] (Table 5).

**Table 5:** Characteristics of studies that evaluated effectiveness and pharmacokinetics of quinine by route of administration (IV vs IM or indirect comparison of IR vs IV and IM vs IR) (Systematic review 3)

Study ID	Age range	Country	IV Quinine Dose	IM Quinine Dose
Waller 1990 (N = 21)	19 months to 8 years	Gambia	N/A	20 mg/kg loading dose, then 10 mg/kg every 12 hours

Pasvol 1991**^‡ ^**(N = 59)	up to 12 years	Kenya	20 mg/kg loading dose, then 10 mg/kg every 12 hours	20 mg/kg loading dose, then 10 mg/kg every 12 hours

Krishna 1991 (N = 120)	1 to 10 years	Ghana	N/A	20 mg/kg loading dose, then 10 mg/kg every 12 hours

Schapira 1993 (N = 104)	6 months to 7 years	Mozambique	20 mg/kg loading dose, then 10 mg/kg every 8 hours	20 mg/kg loading dose, then 10 mg/kg every 8 hours

Winstanley 1993 (N = 22)	7 months to 6.8 years	Kenya	14 mg/kg loading dose, then 10 mg/kg every 12 hours	20 mg/kg loading dose, then 10 mg/kg every 12 hours

Winstanley 1994 (N = 9)	1 to 3 years	Kenya	15 mg/kg loading dose, then 10 mg/kg every 12 hours	N/A

van Hensbroek 1996 (N = 16)	5 to 23 months	The Gambia	20 mg/kg loading dose, then 10 mg/kg every 12 hours	20 mg/kg loading dose, then 10 mg/kg every 12 hours

**Study ID**	**Age**	**Country**	**IR Quinine Dose**	**IV Quinine Dose**

Achan 2007^† ^(N = 110)	6 months to 5 years	Uganda	20 mg/kg base loading dose, then 10 mg/kg every 8 hours, until oral treatment possible	8 mg/kg base every 8 hours, until oral treatment possible

Bareness 1998^† ^(N = 77)	2 to 15 years	Niger	11.8 mg/kg base loading dose, then 8.8 mg/kg every 8 hours for 2 days, then oral treatment with chloroquine	4.7 mg/kg base every 8 hours for 2 days, then oral treatment with chloroquine

**Study ID**	**Age range**	**Country**	**IR Quinine Dose**	**IM Quinine Dose**

Bareness 2001^† ^(N = 58)	2 to 15 years	Niger	17.9 mg/kg base loading dose, then 11.75 mg/kg base every 12 hours	7.5 mg/kg base every 12 hours

^† ^Quinimax formulation which consists of 96% quinine, 2.5% quinidine, 0.68% cinchonine, and 0.67% cinchonidine

^‡^N = 21 patients who received a lower dose of IV administered 10 mg/kg loading dose then 5 mg/kg every 12 hours were excluded from the IV vs IM effectiveness analysis only.

### Systematic review 3a: What is the effectiveness of IV-administered quinine compared to IM-administered quinine in African children with severe malaria?

**Figure 1:**
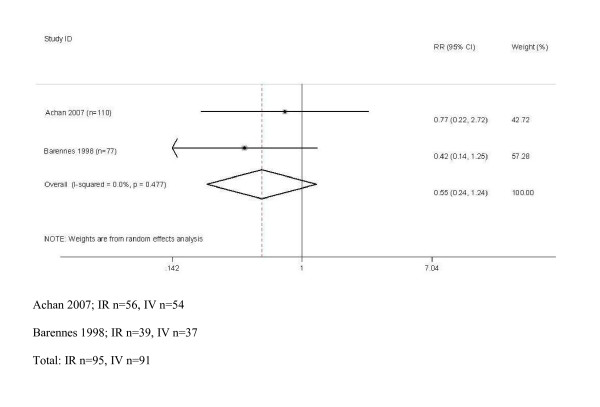
**Meta analysis of comparison of effect of IR and IV administered quinine on mortality in African children**.

Two studies were identified that assessed the comparative effectiveness of quinine route of administration by the IV and IM route when a loading dose of 20 mg/kg was administered, with a maintenance dose of either 10 mg/kg every 12 hours [11] or 10 mg/kg every 8 hours [13]. There was no difference between treatment groups in all the reported outcomes of the individual studies that were all appraised as low quality evidence - risk of death, incidence of neurological sequelae, number of convulsions, coma resolution time, parasite clearance time, fever clearance time, number of hypoglycaemic episodes or haemoglobin levels (see Additional File 3). Further indirect evidence on mortality from 3 studies, deemed to provide very low quality evidence (see Additional File 3), suggested there was no association between risk of death (or any other outcome) and route of administration from the studies that investigated IR quinine vs IV quinine (n = 91 patients) in African children [Odds Ratio (OR) 0.50, 95% CI 0.20 to 1.26; n = 186] [18,19] (Figure 1) or in one study that investigated IR vs IM quinine (OR 0.15, 95% CI 0.01 to 3.28; n = 58) [20]. Overall, therefore, available evidence is low or very low quality to guide decisions on route of quinine administration with the possibility that clinically important risks or benefits exist, but are not recognized.

### Systematic review 3b: What is the pharmacokinetics of IV-administered quinine compared to IM-administered quinine in African children with severe malaria?

Four identified studies evaluated the pharmacokinetics of quinine when administered at a loading dose of 20 mg/kg, followed by a maintenance dose of 10 mg/kg every 12 hours [10-12,16]. One study evaluated the pharmacokinetics of quinine when administered at a loading dose of 20 mg/kg, followed by a maintenance dose of 10 mg/kg every 8 hours [13]. Very low quality evidence showed that there was no difference in the reported pharmacokinetic parameters of quinine in the individual studies - volume of distribution (V_d_), maximum circulating concentration (C_max_), half-life (t_1/2_), area under the curve (AUC) (Table 6).

**Table 6:** Pharmacokinetic parameters of IV and IM administered quinine in African children (Systematic review 3b)

Study ID (sample size)	Evidence Quality	Route of admn	Sample size per arm	Quinine loading dose	Quinine maintenance dose	Infusion time	Cmax (mg/l)	tmax (h)	t_1/2 _(h)	Cl (ml/min/kg)	V_d _(l/kg)	AUC (mg/l/h)
Pasvol 1991 (N = 43)	Low	IV	n = 21	10 mg/kg	5 mg/kg	2 h	9.7 ± 5.3	2.7 ± 1.3	8.9 ± 4.0	1.36 ± 0.58	0.87 ± 0.27	145 ± 61
**Winstanley 1993** **(N = 22)**	**Very** **Low**	**IV**	**n = 12**	**14 mg/kg**	**10 mg/kg bd**	**2 h**	**13.8 ± 3.1**	**-**	**16.4 ± 8.8**	**0.45 ± 0.13**	**0.45 ± 0.10**	
**Winstanley 1994 (N = 9)**	**Very** **Low**	**IV**	**n = 9**	**15 mg/kg**	**10 mg/kg bd**	**2 h**	**17.9 ± 2.9**	**-**	**12.6 ± 3.1**	**0.81 ± 0.24**	**0.75 ± 0.11**	
Pasvol 1991 (N = 43)	Low	IV	n = 18	20 mg/kg	10 mg/kg bd	2 h	15.3 ± 5.5	2.4 ± 1.5	12.5 ± 3.2	1.19 ± 0.29	1.22 ± 0.16	254 ± 86
van Hensbroek 1996 (N = 16)	Very Low	IV	n = 6	20 mg/kg	10 mg/kg bd	4 h	17.48 ± 2.43	-	13.16 ± 6.43	0.51 ± 0.16	1.04 ± 0.21	354 ± 143
**Waller 1990 (N = 21)**	**Very** **Low**	**IM**	**n = 21**	**20 mg/kg**	**10 mg/kg bd**	**N/A**	**15.4 ± 4.5**	**3.23 ± 1.63**	**18.8 ± 8.0**	**0.89 ± 0.81**	**-**	**449 ± 243**
**Pasvol 1991 (N = 43)**	**Low**	**IM**	**n = 20**	**20 mg/kg**	**10 mg/kg bd**	**N/A**	**15.3 ± 7.6**	**2.4 ± 1.7**	**15.7 ± 8.2**	**1.12 ± 0.56**	**1.22 ± 0.32**	**332 ± 254**
**Winstanley 1993 (N = 22)**	**Very** **Low**	**IM**	**n = 10**	**20 mg/kg**	**10 mg/kg bd**	**N/A**	**15.7 ± 3.1**	**1.6 ± 1.7**	**-**	**-**	**-**	**-**
**van Hensbroek 1996 (N = 16)**	**Very** **Low**	**IM**	**n = 10**	**20 mg/kg**	**10 mg/kg bd**	**N/A**	**16.36 ± 3.67**	**1.08 ± 0.37**	**15.31 ± 7.16**	**0.59 ± 0.25**	**1.32 ± 0.49**	**358 ± 164**
**Krishna 2001 (N = 120)**	**Very** **Low**	**IM**	**n = 120**	**20 mg/kg**	**10 mg/kg bd**	**N/A**	**-**	**-**	**19.9 ± 4.4**	**0.05**	**-**	**-**

Legend:

Cmax (mg/l): Maximum circulating concentration

tmax (h): Time at maximum circulating concentration

t_1/2 _(h): Half-life

Cl (ml/min/kg): Clearance

V_d _(l/kg): Volume of distribution

AUC (mg/l/h): Area under the curve.

### Systematic review 4: Is there a link between IV-administered quinine and risk of hypoglycaemia in African children with severe malaria?

Six studies that evaluated the risk of hypoglycaemia in the population and setting of interest to this review were identified [2,21-25] (Table 7). Of these, five studies [21-25] found no effect of IV quinine when given at a slow infusion rate on the number of hypoglycaemic episodes or insulin levels in the children. An association with hypoglycaemia was observed in children, however, when quinine was administered at a high infusion rate. Overall quality of evidence was rated as low or very low to inform decisions on alternative quinine regimens. Recently published high quality evidence, which evaluated the effectiveness of quinine and the artemisinin derivative artesunate in African children up to 15 years of age, however, found 2.8% of patients treated with quinine developed hypoglycaemic episodes, with artesunate treatment associated with a significantly lower risk (OR 0.63, 95% CI 0.43 to 0.91; n = 5,425) [2] (see Additional File 4).

**Table 7:** Characteristics of studies that evaluated risk of hypoglyacemia with IV-infused quinine (Systematic review 4)

Study ID	Evidence quality	Age range	Country	IV Quinine Dose and diluent	Infusion time	**Infusion rate (ml/kg/24 h**)	Glucose Threshold (mmol/l)
Molyneux 1989 (N = 29)	Very low	2 to 9 years	Malawi	10 mg/kg in 5% glucose in half strength Darrow's solution	1 h	80	< 2.2
				10 mg/kg in 5% glucose in half strength Darrow's solution	3 h	80	< 2.2
				20 mg/kg in 5% glucose in half strength Darrow's solution	3 h	80	< 2.2
Okitolonda 1987 (N = 9)*	Very low	6 to 10 years	Zaïre	10 mg/kg in 30 ml saline supplemented with 2.5% glucose every 8 hours	1 h	105	< 2.8
Kawo 1991 (N = 97)	Very low	Up to 7 years	Tanzania	10 mg/kg in 10 ml/kg of 5% glucose every 8 hours	4 h	Not reported	< 2.2
Ogetii 2010 (N = 1237)	Low	Up to 12 years	Kenya	15 mg/kg loading dose in 5% dextrose then 10 mg/kg in 5% dextrose every 12 hours	2 h	Not reported	< 3, < 2.8 and < 2.2
				20 mg/kg loading dose in 5% dextrose then 10 mg/kg in 5% dextrose every 8 hours	4 h	Not reported	< 3, < 2.8 and < 2.2
Taylor 1988 (N = 95)	Very low	7 months to 8 years	Malawi	20 mg/kg loading dose then 10 mg/kg every 8 hours	Loading dose 4 h, then maintenance dose 2 to 8 h	80	< 2.2
Dondorp 2010	High	Up to 15 years	Multi-centre trial in Mozambique, The Gambia, Ghana, Kenya, Tanzania, Nigeria, Uganda, Rwanda and Democratic Republic of Congo	^¥^20 mg/kg loading dose in 5-10 ml/kg of 5% dextrose then 10 mg/kg in 5-10 ml/kg of 5% dextrose every 8 hours	Loading dose 4 h, then maintenance dose 2 to 8 h	Not reported	Not reported

*Data from N = 5 adults were excluded from this analysis

Ogetti 2010 = Retrospective review of case notes. ^¥^Quinine was administered by the IM and IV route.

## Discussion

The WHO malaria treatment guidelines updated in 2010 have introduced the appraisal of studies using the GRADE tool but only for newly acquired evidence since the 2006 guidelines. Of relevance to severe malaria, this encompassed only one topic, the comparison of safety and effectiveness of quinine and artemisinin derivatives in treating severe malaria, with reviews conducted before the recently published large trial on artesunate [2]. Other topics in the WHO 2010 guidance of (i) loading dose of quinine (20 mg/kg) vs. no loading dose, (ii) effectiveness of IM vs IM quinine, (iii) IM artemether vs IV quinine and (iv) safety and efficacy of pre-referral treatment with IR artesunate were reported as narrative summaries of the evidence only, with no transparent assessment or grading of recommendations. Although the new data indicating the superiority of artesunate compared with quinine (see Additional File 5) [2] will change the landscape for guidelines, many African countries are likely to continue to rely on quinine therapy for severe malaria in children for some years to come. Quinine has well known, serious adverse effects and previous and current WHO recommendations (2010) have not used systematic approaches when attempting to appraise the evidence to guide dosing.

Despite more than 30 years of clinical use, only studies of important (not critical) outcomes and only very low or low quality evidence are available to inform most paediatric quinine dosing decisions. Thus, there are no data directly comparing the Kenyan regimen of the last 12 years and only limited data suggesting either the IV or IM routes may be used, although IM quinine is mainly recommended for pre-referral treatment due to the risks of developing an IM injection abscess and other complications when incorrectly administered (Personal Communication, Dr Elizabeth Juma, Head of Division of Malaria Control, Ministry of Public Health and Sanitation). Very limited data also suggest that hypoglycaemia is more likely with faster rates of administration. Only in the case of the use of a loading dose is there moderate quality evidence to guide recommendations, but only for what was considered as non-critical outcomes. These included reduced fever and parasite clearance time with loading doses, but also higher risks of transient hearing loss with higher loading doses [9].

This work thus indicates, historically, how the Kenyan policy on quinine dosing that has been promoted nation-wide for over 10 years, with considerable investments in dissemination of guidelines and in-service training supported by global funds, was based predominantly on low or very low quality evidence by today's standards. The available very low quality evidence does however suggest similar pharmacokinetic parameters for the lower dose Kenyan regimen as the currently recommended WHO regimen. The fact that the 2010 WHO quinine dosing regimens (that are unchanged) are also based on low quality evidence, even for safety, does not provide an obvious rationale for Kenya's change to adopt the WHO guidance. While it is considered entirely appropriate that other factors are considered in making recommendations based on evidence [3,26], this process is rarely transparent, as is the case with Kenya's change of its quinine regimen.

The emergent message from the series of systematic reviews we conducted is the paucity of high quality evidence that may inform the dose, route of administration and safety of treatment of African children diagnosed with severe malaria with quinine, notwithstanding that quinine has been the standard treatment of severe malaria for decades (Table 8). This contrasts starkly with the evidence available to inform policy for children with non-severe malaria. Here international efforts and funding have supported over fifty randomized controlled trials in recent years [27]. Despite huge investments to tackle malaria over the last decade or more [28] it is clear therefore that severe malaria in African children has remained a neglected disease. In conducting these systematic reviews we found the GRADE approach a very useful tool for making the presence of only low quality evidence explicit. This paper postulates that the wider use of this or other similar approaches can help build advocacy efforts for a greater research focus on priority areas in paediatric therapeutics in African and other low-income settings.

**Table 8:** Summary of quinine systematic reviews in the treatment of severe malaria in African children and recommendations for evidence-informed clinical practice

	Clinical Issue	Recommendation	Evidence quality/strength of recommendation
**Systematic review 1: Is there a value in administration of a loading dose of quinine in African children with severe malaria?**	Quinine loading dose [9]	Loading dose results in faster clearance of malaria parasites from the blood stream and thus faster clearance of fever and thus loading dose should be administered, with monitoring and patient/care giver support for episodes of partial hearing loss adverse event	Moderate
**Systematic review 3a: What is the effectiveness of IV-administered quinine compared to IM-administered quinine in African children with severe malaria**	Quinine route of administration: IV vs IM [11,13,18-20]	The clinical outcome of IV-administered quinine vs. IM-administered quinine is equivocal in the treatment of African children with severe malaria. However, due to reported side effects with IM route, such as risk of abscess and pain, the IV route is preferred.	Low to very low
**Systematic review 3b: What is the pharmacokinetics of IV-administered quinine compared to IM-administered quinine in African children with severe malaria**	Quinine paediatric dose [10-12,14-16]	Pharmacokinetic profile of the old and new Kenyan dosing regimen are similar	Low
**Systematic review 4: Is there a link between IV-administered quinine and risk of hypoglycaemia in African children with severe malaria?**	Quinine and risk of hypoglycaemia [2,21-25]	The risk of hypoglycaemia with quinine treatment in African children with severe malaria may be countered by administering the quinine at a low infusion rate. Glucose levels should be monitored due to hypoglycaemic risk that occurs due to the disease and/or quinine treatment, and should be treated with glucose infusion	Low to high
**AQUAMAT Findings**	Quinine vs artemisinin derivatives [2]	Artesunate is a superior treatment to quinine for African children with severe malaria and should be strongly considered for implementation as a first line treatment, taking contextual factors such as cost-effectiveness into account	High

The recent findings of the large multicentre trial demonstrating superiority of artesunate over quinine for severe malaria in African children [2] provide a clear argument for a change in approach on the continent. These data demonstrate how poor the foundational basis was for the use of quinine and should help argue that the practical difficulties of introducing artesunate should be overcome as soon as possible. Further, this work supports that efforts to introduce explicit methods of examining the evidence base for treatment of children in low-income settings should be strongly supported.

## Competing interests

The authors declare that they have no competing interests.

## Authors' contributions

ME conceived of the topics for the systematic reviews. NM developed the methodology and conducted the reviews with assistance from NO. NM drafted the manuscript and ME and NO contributed to it. All authors read and approved the final manuscript.

## Supplementary Material

Additional file 1 **Flow diagrams of the study selection process for each of the systematic review questions**. A series of flow diagrams depicting the number of studies found in the literature search, the number excluded during assessment and the final number included in the systematic reviews. Click here for file

Additional file 2 **GRADE Table for studies included in systematic review 1: Is there a value in administration of a loading dose of quinine in African children with severe malaria?**. Critical appraisal and outcome data using the GRADE tool for Lesi 2004 Cochrane systematic review. Click here for file

Additional file 3 **GRADE Tables for studies included in systematic review 3a: What is the effectiveness of IV-administered quinine compared to IM-administered quinine in African children with severe malaria?**. Critical appraisal and outcome data using the GRADE tool for Schapira 1993, Pasvol 1991 (direct comparisons) and included studies from the Eisenhut 2009 Cochrane systematic review - Achan 2007; Barennes 1998; Barennes 2001(indirect comparisons) Click here for file

Additional file 4 **GRADE Table for studies included in systematic review 4: Is there a link between IV-administered quinine and risk of hypoglycaemia in African children with severe malaria?**. Critical appraisal and outcome data using the GRADE tool for Ogetti 2010 and Dondorp 2010. Click here for file

Additional file 5 **GRADE Table for the African multi-centre trial (AQUAMAT)**. Critical appraisal and mortality data using the GRADE tool for Dondorp 2010 and Eltahir 2010. Click here for file
