# Supplementary material for: Treatment of African children with severe malaria - towards evidence-informed clinical practice using GRADE
Source: Malar J. 2011 Jul 21;10:201. doi: 10.1186/1475-2875-10-201 (PMC3152530; doi:10.1186/1475-2875-10-201)

Additional File 1

Flow diagrams of the study selection process for each of the systematic reviews

Systematic review 1: Is there a value in administration of a loading dose of quinine in African children with severe malaria?

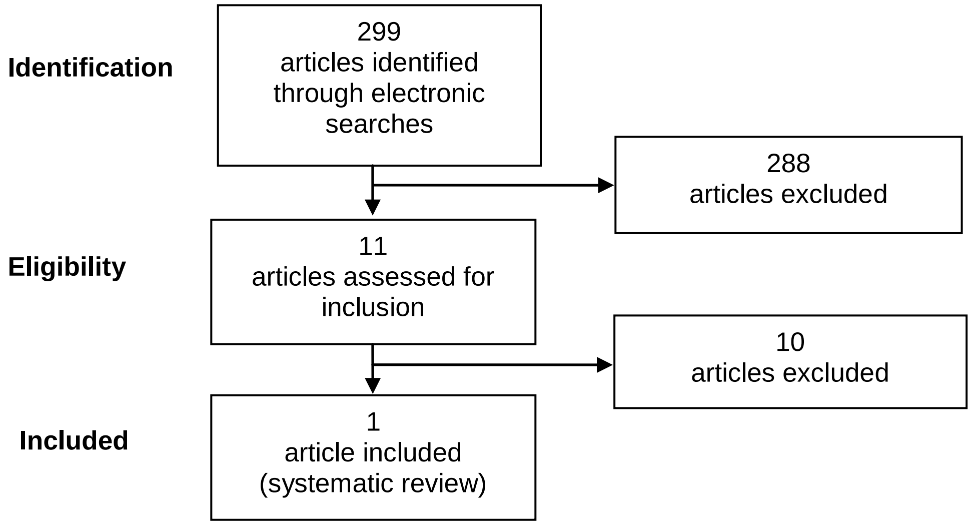

Systematic review 2: Should Kenya change its recommendation for treatment of severe malaria in children under 5 of 15mg /kg loading dose followed by 10mg /kg every 12 hours and replace it with the WHO recommended regimen of 20mg /kg loading dose followed by 10mg /kg every 8 hours?

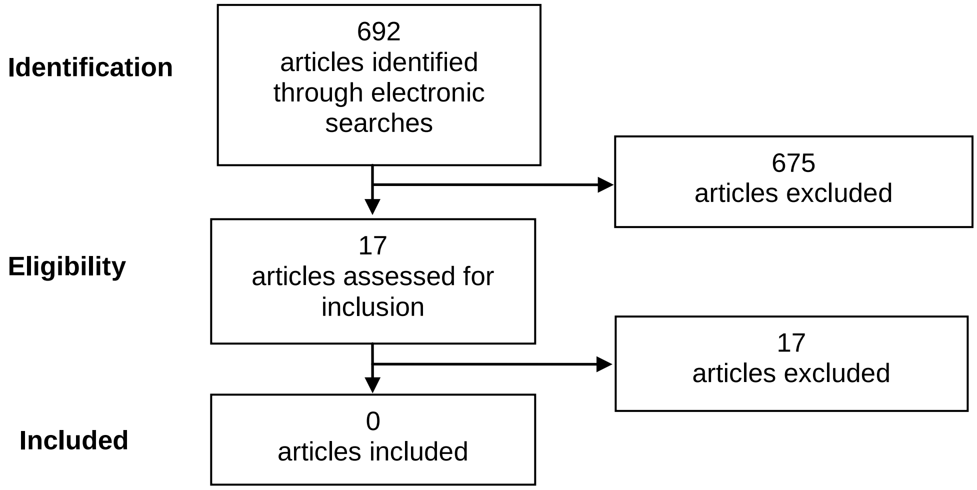

Systematic review 3: What are the pharmacokinetics and effectiveness of IV-administered quinine compared to IM-administered quinine in African children with severe malaria?

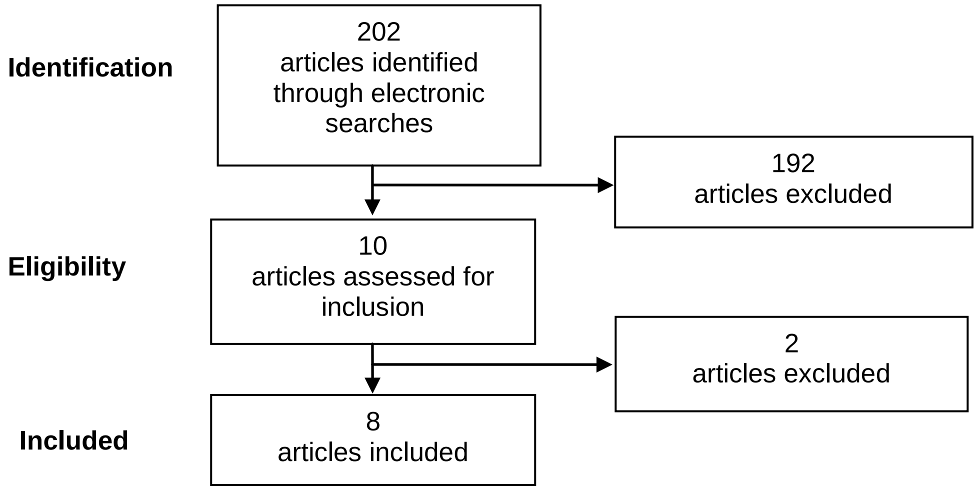

Systematic review 4: Is there a link between IV-administered quinine and risk of hypoglycaemia in African children with severe malaria?

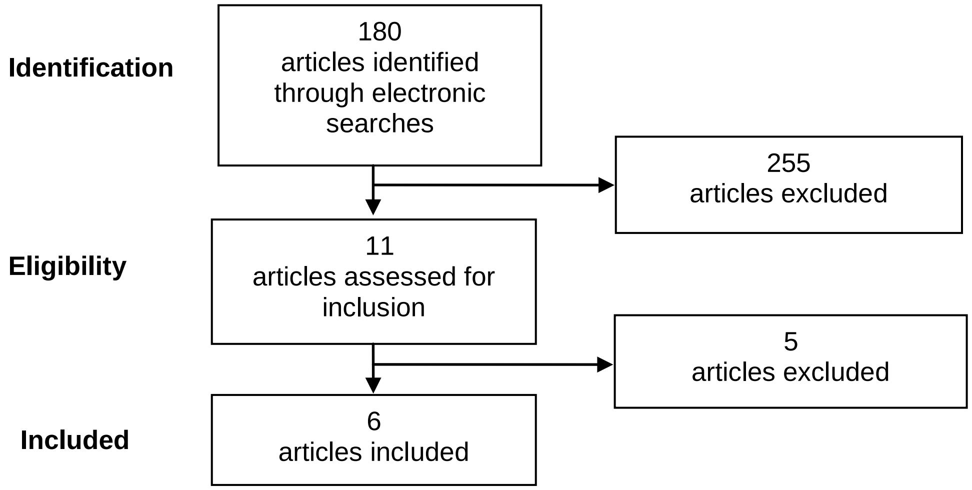

Supplement: Additional file 1 — Flow diagrams of the study selection process for each of the systematic review questions. A series of flow diagrams depicting the number of studies found in the literature search, the number excluded during assessment and the final number included in the systematic reviews. [file 1475-2875-10-201-S1.PDF]
