# Supplementary material for: Treatment of African children with severe malaria - towards evidence-informed clinical practice using GRADE
Source: Malar J. 2011 Jul 21;10:201. doi: 10.1186/1475-2875-10-201 (PMC3152530; doi:10.1186/1475-2875-10-201)
Supplement: Additional file 2 — GRADE Table for studies included in systematic review 1: Is there a value in administration of a loading dose of quinine in African children with severe malaria?. Critical appraisal and outcome data using the GRADE tool for Lesi 2004 Cochrane systematic review. [file 1475-2875-10-201-S2.PDF]

Additional File 2

GRADE Table for studies included in systematic review 1: Is there a value in administration of a loading dose of quinine in African children with severe malaria?

Bibliography: Lesi A, Meremikwu M: **High first dose quinine regimen for treating severe malaria**. *Cochrane Database Syst Rev* 2004, **3**:CD003341.

Setting: Africa

Quality assessment							Summary of findings					Importance
							No of patients		Effect		Quality	
No of studies	Design	Limitations	Inconsistency	Indirectness	Imprecision	Other considerations	20mg/kg loading dose then 10mg/kg twice or three times daily maintenance dose	10mg/kg uniform dose twice or three times daily	Relative (95% CI)	Absolute		
**Death**												
3	randomised trials	serious[1]	no serious inconsistency	no serious indirectness	no serious imprecision	none	4/70 (5.7%)	7/74 (9.5%)	RR 0.62 (0.19 to 2.04)	36 fewer per 1000 (from 77 fewer to 98 more)	⊕⊕⊕O MODERATE	CRITICAL
**Coma recovery time (measured with: hours; Better indicated by lower values)**												
2	randomised trials	serious[2]	no serious inconsistency	no serious indirectness	no serious imprecision	none	48	51	-	MD 5.17 higher (1.14 lower to 11.47 higher)	⊕⊕⊕O MODERATE	CRITICAL
**Convulsions**												
1	randomised trials	serious[3]	no serious inconsistency	no serious indirectness	no serious imprecision	none	5/18 (27.8%)	8/21 (38.1%)<br>0%	RR 0.73 (0.29 to 1.84)	103 fewer per 1000 (from 270 fewer to 320 more)<br>0 fewer per 1000 (from 0 fewer to 0 more)	⊕⊕⊕O MODERATE	CRITICAL
**Fever clearance time (measured with: hours; Better indicated by lower values)**												
2	randomised trials	serious[4]	no serious inconsistency	no serious indirectness	no serious imprecision	none	33	35	-	MD 11.11 lower (20.04 to 2.18 lower)	⊕⊕⊕O MODERATE	IMPORTANT

**Parasite clearance time (measured with: hours; Better indicated by lower values)**												
2	randomised trials	serious	no serious inconsistency	no serious indirectness	no serious imprecision	none	34	33	-	MD 7.44 lower (13.24 to 1.64 lower)	⊕⊕⊕O MODERATE	IMPORTANT
**Number with asexual parasitaemia at 24 hours (follow-up mean 24 hours; Better indicated by lower values)**												
1	randomised trials	serious[5]	no serious inconsistency	no serious indirectness	serious[6]	none	35	37	-	MD 1.27 higher (0.87 to 1.84 higher)	⊕⊕OO LOW	IMPORTANT
**Number with asexual parasitaemia at 48 hours (follow-up mean 48 hours; Better indicated by lower values)**												
1	randomised trials	serious[6]	no serious inconsistency	no serious indirectness	serious[6]	none	35	37	-	MD 0.08 higher (0 to 1.39 higher)	⊕⊕OO LOW	IMPORTANT
**Neurological sequalae**												
2	randomised trials	serious	no serious inconsistency	no serious indirectness	no serious imprecision	none	2/53 (3.8%)	4/58 (6.9%)	RR 0.56 (0.11 to 2.9)	30 fewer per 1000 (from 61 fewer to 131 more)	⊕⊕⊕O MODERATE	CRITICAL
								0%		0 fewer per 1000 (from 0 fewer to 0 more)		
**Adverse events: hypoglycaemia**												
2	randomised trials	serious	no serious inconsistency	no serious indirectness	no serious imprecision	none	4/35 (11.4%)	3/37 (8.1%)	RR 1.39 (0.32 to 6)	32 more per 1000 (from 55 fewer to 405 more)	⊕⊕⊕O MODERATE	IMPORTANT
								0%		0 more per 1000 (from 0 fewer to 0 more)		
**Adverse events: tinnitus**												
1	randomised trials	serious	no serious inconsistency	no serious indirectness	no serious imprecision	none	3/17 (17.6%)	1/16 (6.3%)	RR 2.82 (0.33 to 24.43)	114 more per 1000 (from 42 fewer to 1464 more)	⊕⊕⊕O MODERATE	IMPORTANT
								0%		0 more per 1000 (from 0 fewer to 0 more)		
**Adverse events: hearing loss**												
1	randomised trials	serious	no serious inconsistency	no serious indirectness	no serious imprecision	none	10/17 (58.8%)	3/16 (18.8%)	RR 3.14 (1.05 to 9.38)	401 more per 1000 (from 9 more to 1571 more)	⊕⊕⊕O MODERATE	IMPORTANT
								0%		0 more per 1000 (from 0 more to 0 more)		
**Adverse events: hypotension**												
1	randomised	serious	no serious	no serious	no serious	none	16/17 (94.1%)	15/16	RR 1.00	0 fewer per		IMPORTANT

	trials		inconsistency	indirectness	imprecision			(93.8%)	(0.13 to 75.24)	1000 (from 816 fewer to 69600 more)	⊕⊕⊕O MODERATE	
								0%		0 fewer per 1000 (from 0 fewer to 0 more)		
**Adverse events: arrhythmia (prolonged QT interval)**												
1	randomised trials	serious	no serious inconsistency	no serious indirectness	serious	none	1/35 (2.9%)	0/37 (0%)	RR 3.17 (0.13 to 75.24)	0 more per 1000 (from 0 fewer to 0 more)	⊕⊕OO LOW	IMPORTANT
								0%		0 more per 1000 (from 0 fewer to 0 more)		

[1] Randomisation unclear for Assimadi 2001; allocation concealment explicit only in 1 study (Pasvol 1991); losses to follow up unclear in 3 studies and inadequate in Pasvol 1991

[2] Randomisation unclear in Assimadi 2002

[3] Pasvol 1991 - good randomisation & allocation concealment; no blinding; inadequate loss to follow up information

[4] Pasvol 1991 & Tombe 1992 both had good randomisation but only Pasvol 1991 had good allocation concealment

[5] Assimadi 2002 - unclear randomisation reported, no allocation concealment and no blinding thus a possibility of bias in findings
