# Supplementary material for: Treatment of African children with severe malaria - towards evidence-informed clinical practice using GRADE
Source: Malar J. 2011 Jul 21;10:201. doi: 10.1186/1475-2875-10-201 (PMC3152530; doi:10.1186/1475-2875-10-201)
Supplement: Additional file 3 — GRADE Tables for studies included in systematic review 3a: What is the effectiveness of IV-administered quinine compared to IM-administered quinine in African children with severe malaria?. Critical appraisal and outcome data using the GRADE tool for Schapira 1993, Pasvol 1991 (direct comparisons) and included studies from the Eisenhut 2009 Cochrane systematic review - Achan 2007; Barennes 1998; Barennes 2001(indirect comparisons) [file 1475-2875-10-201-S3.PDF]

Additional File 3

GRADE Tables for studies included in systematic review 3a: What is the effectiveness of IV-administered quinine compared to IM-administered quinine in African children with severe malaria?

Bibliography: Schapira A, Solomon T, Julien M, Macome A, Parmar N, Ruas I, Simao F, Streat E, Betschart B: **Comparison of intramuscular and intravenous quinine for the treatment of severe and complicated malaria in children**. *Trans R Soc Trop Med Hyg* 1993, **87**:299-302.

Setting: Africa

Quality assessment							Summary of findings					Importance
							No of patients		Effect		Quality	
No of studies	Design	Limitations	Inconsistency	Indirectness	Imprecision	Other considerations	IV quinine	IM quinine	Relative (95% CI)	Absolute		
**Death (follow-up mean 21 days)**												
1	randomised trials	serious[1]	no serious inconsistency	no serious indirectness	serious[2]	none	8/47 (17%)	4/57 (7%)	RR 2.43 (0.78 to 7.56)	100 more per 1000 (from 15 fewer to 460 more)	⊕⊕OO LOW	CRITICAL
								0%		0 more per 1000 (from 0 fewer to 0 more)		
**neurological sequelae (follow-up mean 21 days)**												
1	randomised trials	serious[1]	no serious inconsistency	serious	serious[2]	none	1/38 (2.6%)	3/52 (5.8%)	RR 0.47 (0.05 to 4.35)	31 fewer per 1000 (from 55 fewer to 193 more)	⊕⊕OO LOW	CRITICAL
								0%		0 fewer per 1000 (from 0 fewer to 0 more)		
**death + neurological sequelae (follow-up mean 21 days)**												

1	randomised trials	serious[1]	no serious inconsistency	no serious indirectness	serious[2]	none	0/0 (0%)	0/0 (0%)	RR 1.58 (0 to 0)	0 more per 1000 (from 0 fewer to 0 fewer)	⊕⊕OO LOW	CRITICAL
								0%		0 more per 1000 (from 0 fewer to 0 fewer)		
**coma clearance time (follow-up mean 21 days; measured with: hours; Better indicated by lower values)**												
1	randomised trials	serious[1]	no serious inconsistency	no serious indirectness	serious[2]	none	47	57	-	MD 1.7 lower (18.9 lower to 15.5 higher)	⊕⊕OO LOW	CRITICAL
**temperature (fever) clearance time (follow-up mean 48 hours; measured with: hours; Better indicated by lower values)**												
1	randomised trials	serious[1]	no serious inconsistency	no serious indirectness	serious[2]	none	47	57	-	MD 4.3 lower (21.3 lower to 12.7 higher)	⊕⊕OO LOW	IMPORTANT
**asexual parasitaemia clearance time (follow-up mean 48 hours ; measured with: hours; Better indicated by lower values)**												
1	randomised trials	serious[1]	no serious inconsistency	no serious indirectness	serious[2]	none	0	0	-	MD 0.7 higher (7.6 lower to 9.0 higher)	⊕⊕OO LOW	IMPORTANT
**plasma glucose days 1 to 4 (follow-up mean 21 days; measured with: mM; Better indicated by higher values)**												
1	randomised trials	serious[1]	no serious inconsistency	serious	serious[2]	none	47	57	-	MD 0.39 higher (0.2 to 0.6 higher)	⊕⊕OO LOW	IMPORTANT
**Hb on day 7 (follow-up mean 7 days; measured with: mM; Better indicated by higher values)**												
1	randomised trials	serious[1]	no serious inconsistency	no serious indirectness	serious[2]	none	47	57	-	MD 3.6 lower (9.9 lower to 2.7 higher)	⊕⊕OO LOW	IMPORTANT

[1] no allocation concealment. lab personnel but not clinical personnel were blinded.
[2] small sample size - likely not powered to detect differences

Bibliography: Pasvol G, Newton CR, Winstanley PA, Watkins WM, Peshu NM, Were JB, Marsh K, Warrell DA: **Quinine treatment of severe falciparum malaria in African children: a randomized comparison of three regimens**. *Am J Trop Med Hyg* 1991, **45**:702-713.

Setting: Africa

Quality assessment							Summary of findings					Importance
							No of patients		Effect		Quality	
No of studies	Design	Limitations	Inconsistency	Indirectness	Imprecision	Other considerations	IV quinine	IM quinine	Relative (95% CI)	Absolute		
**Death**												
1	randomised trials	serious[1]	no serious inconsistency	no serious indirectness	serious[2]	none	1/18 (5.6%)	3/20 (15%)	RR 0.37 (0.04 to 3.24)	95 fewer per 1000 (from 144 fewer to 336 more)	⊕⊕OO LOW	CRITICAL
								0%		0 fewer per 1000 (from 0 fewer to 0 more)		
**neurological sequelae**												
1	randomised trials	serious	no serious inconsistency	no serious indirectness	serious[2]	none	1/18 (5.6%)	2/20 (10%)	RR 0.56 (0.05 to 5.62)	44 fewer per 1000 (from 95 fewer to 462 more)	⊕⊕OO LOW	CRITICAL
								0%		0 fewer per 1000 (from 0 fewer to 0 more)		
**number of convulsions**												
1	randomised trials	serious	no serious inconsistency	no serious indirectness	serious[2]	none	5/18 (27.8%)	8/20 (40%)	RR 0.69 (0.28 to 1.73)	124 fewer per 1000 (from 288 fewer to 292 more)	⊕⊕OO LOW	CRITICAL
								0%		0 fewer		

										per 1000 (from 0 fewer to 0 more)		
**recovery of consciousness (coma clearance) time (measured with: hours ; Better indicated by lower values)**												
1	randomised trials	serious	no serious inconsistency	no serious indirectness	serious[2]	none	0	0	-	MD 4 lower (16.8 lower to 8.8 higher)	⊕⊕OO LOW	CRITICAL
**parasite clearance time (measured with: hours ; Better indicated by lower values)**												
1	randomised trials	serious	no serious inconsistency	no serious indirectness	serious[2]	none	0	0	-	MD 1 higher (10.6 lower to 12.6 higher)	⊕⊕OO LOW	IMPORTANT
**time to defervescence (measured with: hours ; Better indicated by lower values)**												
1	randomised trials	serious	no serious inconsistency	no serious indirectness	serious[2]	none	0	0	-	MD 12 lower (24 lower to 0 higher)	⊕⊕OO LOW	IMPORTANT

[1] randomisation method not specified. no information on blinding/concealment
[2] small sample sizes - likely not powered to detect differences

Bibliography: Eisenhut M, Omari AA: **Intrarectal quinine versus intravenous or intramuscular quinine for treating Plasmodium falciparum malaria**. *Cochrane Database Syst Rev* 2009, **21**:CD004009.

Setting: Africa

No of studies	Design	Limitations	Inconsistency	Indirectness	Imprecision	Other considerations	IV quinine	IM quinine	Relative (95% CI)	Absolute	Quality	Importance
**Quality assessment**							**Summary of findings**					
							**No of patients**		**Effect**			
**death IR vs IV**												
2	randomised trials	serious[1]	no serious inconsistency	serious[2]	serious[3]	none	8/95 (8.4%)	14/91 (15.4%)	RR 0.55 (0.24 to 1.24)	69 fewer per 1000 (from 117 fewer to 37 more)	⊕OOO VERY LOW	CRITICAL
								0%		0 fewer per 1000 (from 0 fewer to 0 more)		
**death IR vs IM**												
1	randomised trials	serious[1]	no serious inconsistency	serious[2]	serious[3]	none	0/32 (0%)	2/26 (7.7%)	RR 0 (0 to 0)	77 fewer per 1000 (from 77 fewer to 77 fewer)	⊕OOO VERY LOW	CRITICAL
								0%		0 fewer per 1000 (from 0 fewer to 0 fewer)		

[1] no blinding or allocation concealment reported, except for Achan 2007

[2] indirect comparison for IR vs IM and IR vs IV studies may be biased due to small sample sizes and different inclusion criteria in the three studies small sample sizes therefore not powered to detect statistical significant differences in the reported outcomes

[3] small sample sizes therefore not powered to detect statistical significant differences in the reported outcomes
