# Supplementary material for: Treatment of African children with severe malaria - towards evidence-informed clinical practice using GRADE
Source: Malar J. 2011 Jul 21;10:201. doi: 10.1186/1475-2875-10-201 (PMC3152530; doi:10.1186/1475-2875-10-201)
Supplement: Additional file 4 — GRADE Table for studies included in systematic review 4: Is there a link between IV-administered quinine and risk of hypoglycaemia in African children with severe malaria?. Critical appraisal and outcome data using the GRADE tool for Ogetti 2010 and Dondorp 2010. [file 1475-2875-10-201-S4.PDF]

Additional File 4

GRADE Table for studies included in systematic review 4: Is there a link between IV-administered quinine and risk of hypoglycaemia in African children with severe malaria?

Bibliography: Dondorp AM, Fanello CI, Hendriksen IC, Gomes E, Seni A, Chhaganlal KD, Bojang K, Olaosebikan R, Anunobi N, Maitland K, Kivaya E, Agbenyega T, Nguah SB, Evans J, Gesase S, Kahabuka C, Mtove G, Nadjm B, Deen J, Mwanga-Amumpaire J, Nansumba M, Karema C, Umulisa N, Uwimana A, Mokuolu OA, Adedoyin OT, Johnson WBR, Tshefu AK, Onyamboko MA, Sakulthaew T, Ngum WP, Silamut K, Stepniewska K, Woodrow CJ, Bethell D, Wills B, Oneko M, Peto TE, von Seidlein L, Day NPJ, White NJ, for the AQUAMAT group: **Artesunate versus quinine in the treatment of severe falciparum malaria in African children (AQUAMAT): an open-label, randomised trial**. *Lancet* 2010, **376**:1647-1657.

Setting: Africa

Quality assessment							Summary of findings					Importance
							No of patients		Effect		Quality	
No of studies	Design	Limitations	Inconsistency	Indirectness	Imprecision	Other considerations	quinine	artesunate	Relative (95% CI)	Absolute		
**hypoglycaemia**												
1	randomised trials	no serious limitations	no serious inconsistency	no serious indirectness	no serious imprecision	none	75/2713 (2.8%)	48/2712 (1.8%)	OR 0.63 (0.43 to 0.91)	6 fewer per 1000 (from 2 fewer to 10 fewer)	⊕⊕⊕⊕ HIGH	IMPORTANT

Bibliography: Ogetii GN, Akech S, Jemutai J, Boga M, Kivaya E, Fegan G, Maitland K: **Hypoglycaemia in severe malaria, clinical associations and relationship to quinine dosage**. *BMC Infect Dis*, **10**:334.

Setting: Africa

Quality assessment							Summary of findings					
							No of patients		Effect		Quality	Importance
**No of studies**	**Design**	**Limitations**	**Inconsistency**	**Indirectness**	**Imprecision**	**Other considerations**	**quinine regimen of 15mg /kg loading dose followed by 10mg /kg bd**	**20mg /kg loading dose followed by 10mg /kg tds**	**Relative (95% CI)**	**Absolute**		
**death: overall per cohort**												
1	observational studies	serious[1]	no serious inconsistency	no serious indirectness	no serious imprecision	reduced effect for RR >> 1 or RR << 1	101/954 (10.6%)	25/283 (8.8%)	RR 0 (0 to 0)	88 fewer per 1000 (from 88 fewer to 88 fewer)	⊕⊕OO LOW	CRITICAL
								0%		0 fewer per 1000 (from 0 fewer to 0 fewer)		
**overall death: in all hypoglycaemic cases vs cases that remained euglycaemic (<=3 mmmol/l)**												
1	observational studies	serious[1]	no serious inconsistency	no serious indirectness	no serious imprecision	reduced effect for RR >> 1 or RR << 1	42/187 (22.5%)	81/1050 (7.7%)	RR 2.55 (1.91 to 3.41)	120 more per 1000 (from 70 more to 186 more)	⊕⊕OO LOW	CRITICAL
**all hypoglycaemia frequency post-admission (<= 3 mmol/l) (follow-up 3-4 years; <=3.0 mmol/l)**												
1	observational studies	serious[1]	no serious inconsistency	no serious indirectness	no serious imprecision	reduced effect for RR >> 1 or RR << 1[2]	145/954 (15.2%)	42/283 (14.8%)	not pooled	not pooled	⊕⊕OO LOW	IMPORTANT
								0%		not pooled		

**all post-admission hypoglycaemic that were euglycaemic at baseline (follow-up 3-4 years; <= 3 mmols/l)**												
1	observational studies	serious[1]	no serious inconsistency	no serious indirectness	no serious imprecision	reduced effect for RR >> 1 or RR << 1	103/145 (71%)	27/42 (64.3%)	RR 0 (0 to 0)	643 fewer per 1000 (from 643 fewer to 643 fewer)	⊕⊕OO LOW	IMPORTANT
**timing of 1st post-admission hypo episode: 0-24 hours**												
1	observational studies	serious[1]	no serious inconsistency	no serious indirectness	no serious imprecision	reduced effect for RR >> 1 or RR << 1	118/145 (81.4%)	30/42 (71.4%)	RR 0 (0 to 0)	714 fewer per 1000 (from 714 fewer to 714 fewer)	⊕⊕OO LOW	IMPORTANT
								0%		0 fewer per 1000 (from 0 fewer to 0 fewer)		
**timing of 1st post-admission hypo episode: after 24 hours**												
1	observational studies	serious[1]	no serious inconsistency	no serious indirectness	no serious imprecision	reduced effect for RR >> 1 or RR << 1	19/145 (13.1%)	4/42 (9.5%)	RR 0 (0 to 0)	95 fewer per 1000 (from 95 fewer to 95 fewer)	⊕⊕OO LOW	IMPORTANT
								0%		0 fewer per 1000 (from 0 fewer to 0 fewer)		
**timing of 1st post-admission hypo episode: after 48 hours**												
1	observational studies	serious[1]	no serious inconsistency	no serious indirectness	no serious imprecision	reduced effect for RR >> 1 or RR << 1	8/145 (5.5%)	4/42 (9.5%)	RR 0 (0 to 0)	95 fewer per 1000 (from 95 fewer to 95 fewer)	⊕⊕OO LOW	IMPORTANT
								0%		0 fewer per 1000 (from 0 fewer to 0 fewer)		
**severe hypoglycaemia frequency post-admission (<2.2 mmol/l)**												
1	observational	serious[1]	no serious	no serious	no serious	reduced effect	74/954	13/283	RR 0 (0	46 fewer	⊕⊕OO	IMPORTANT

	studies		inconsistency	indirectness	imprecision	for RR >> 1 or RR << 1	(7.8%)	(4.6%)	to 0)	per 1000 (from 46 fewer to 46 fewer)	LOW	
								0%		0 fewer per 1000 (from 0 fewer to 0 fewer)		
**moderate hypoglycaemia frequency post-admission (<2.5 mmol/l)**												
1	observational studies	serious[1]	no serious inconsistency	no serious indirectness	no serious imprecision	reduced effect for RR >> 1 or RR << 1	103/954 (10.8%)	22/283 (7.8%)	RR 0 (0 to 0)	78 fewer per 1000 (from 78 fewer to 78 fewer)	⊕⊕OO LOW	IMPORTANT
								0%		0 fewer per 1000 (from 0 fewer to 0 fewer)		
								0%		0 more per 1000 (from 0 more to 0 more)		

[1] retrospective case notes review study. the two cohorts were not identical in overall disease severity indices + rate of quinine administration was not identical between the cohorts.

[2] very sick patients as all in HDU (larger benefit seen from sicker patients). also discrepancy in rate of quinine administration implies the later cohort may have had decreased hypoglycaemic events due to the slower infusion, so effect may be due to this as opposed to dosing - potentially could be higher rate of events which is masked by reduced infusion rate
