# Supplementary material for: Treatment of African children with severe malaria - towards evidence-informed clinical practice using GRADE
Source: Malar J. 2011 Jul 21;10:201. doi: 10.1186/1475-2875-10-201 (PMC3152530; doi:10.1186/1475-2875-10-201)
Supplement: Additional file 5 — GRADE Table for the African multi-centre trial (AQUAMAT). Critical appraisal and mortality data using the GRADE tool for Dondorp 2010 and Eltahir 2010. [file 1475-2875-10-201-S5.PDF]

Additional File 5

GRADE Table for the African multi-centre trial (AQUAMAT)

Bibliography: Dondorp AM, Fanello CI, Hendriksen IC, Gomes E, Seni A, Chhaganlal KD, Bojang K, Olaosebikan R, Anunobi N, Maitland K, Kivaya E, Agbenyega T, Nguah SB, Evans J, Gesase S, Kahabuka C, Mtove G, Nadjm B, Deen J, Mwanga-Amumpaire J, Nansumba M, Karema C, Umulisa N, Uwimana A, Mokuolu OA, Adedoyin OT, Johnson WBR, Tshefu AK, Onyamboko MA, Sakulthaew T, Ngum WP, Silamut K, Stepniewska K, Woodrow CJ, Bethell D, Wills B, Oneko M, Peto TE, von Seidlein L, Day NPJ, White NJ, for the AQUAMAT group: **Artesunate versus quinine in the treatment of severe falciparum malaria in African children (AQUAMAT): an open-label, randomised trial**. *Lancet* 2010, **376**:1647-1657.

Setting: Africa

Quality assessment							Summary of findings					Importance
							No of patients		Effect		Quality	
No of studies	Design	Limitations	Inconsistency	Indirectness	Imprecision	Other considerations	quinine	artesunate	Relative (95% CI)	Absolute		
mortality												
2	randomised trials	no serious limitations	no serious inconsistency	no serious indirectness	no serious imprecision	none	299/2746 (10.9%)	231/2745 (8.4%)	OR 0.75 (0.59 to 0.95)	20 fewer per 1000 (from 4 fewer to 33 fewer)	⊕⊕⊕⊕ HIGH	CRITICAL
								0%		0 fewer per 1000 (from 0 fewer to 0 fewer)		
